# A Low Permeability Microfluidic Blood-Brain Barrier Platform with Direct Contact between Perfusable Vascular Network and Astrocytes

**DOI:** 10.1038/s41598-017-07416-0

**Published:** 2017-08-14

**Authors:** Seokyoung Bang, Seung-Ryeol Lee, Jihoon Ko, Kyungmin Son, Dongha Tahk, Jungho Ahn, Changkyun Im, Noo Li Jeon

**Affiliations:** 1 Division of WCU (World Class University) Multiscale Mechanical Design, School of Mechanical and Aerospace Engineering, Seoul National University, Seoul, South Korea; 2 BK21 Plus Transformative Training Program for Creative Mechanical Engineers, Seoul National University, Seoul, South Korea; 3 Institute of Advanced Machinery and Design, Seoul National University, Seoul, South Korea

## Abstract

A novel three dimensional blood brain barrier (BBB) platform was developed by independently supplying different types of media to separate cell types within a single device. One channel (vascular channel, VC) is connected to the inner lumen of the vascular network while the other supplies media to the neural cells (neural channel, NC). Compared to co-cultures supplied with only one type of medium (or 1:1 mixture), best barrier properties and viability were obtained with culturing HUVECs with endothelial growth medium (EGM) and neural cells with neurobasal medium supplemented with fetal bovine serum (NBMFBS) independently. The measured vascular network permeability were comparable to reported *in vivo* values (20 kDa FITC-dextran, 0.45 ± 0.11 × 10^−6^ cm/s; 70 kDa FITC-dextran, 0.36 ± 0.05 × 10^−6^ cm/s) and a higher degree of neurovascular interfacing (astrocytic contact with the vascular network, GFAP-CD31 stain overlap) and presence of synapses (stained with synaptophysin). The BBB platform can dependably imitate the perivascular network morphology and synaptic structures characteristic of the NVU. This microfluidic BBB model can find applications in screening pharmaceuticals that target the brain for in neurodegenerative diseases.

## Introduction

**Figure 1:**
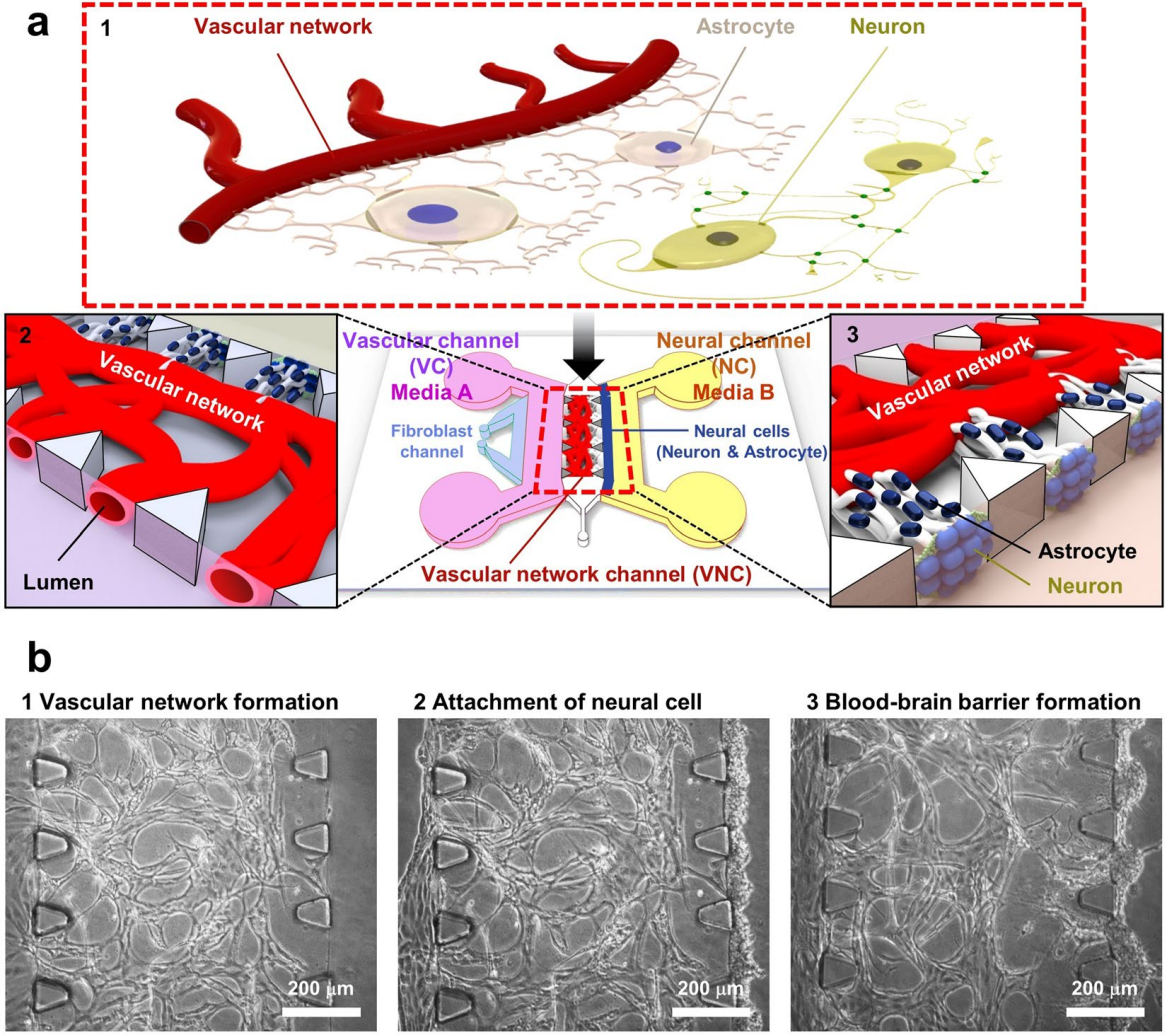
Microfluidic platform for neurovascular unit (NVU) including blood-brain barrier (BBB). (**a**) The neurovascular unit includes a highly selectively permeable vascular feature called the blood-brain barrier which is composed of a complex network of capillaries and astrocytes. The astrocytes are directly interfaced with the capillaries through astrocytic endfeet, which anchor the neurons to the vascular network. Adjacent to the BBB are neural circuits composed of synaptic neural networks. (1) Proposed *in vitro* 3D NVU platform. The *in vitro* NVU platform consists of an engineered BBB co-culture of astrocytes integrated into a vascular network, and a neuronal network “interior” to the BBB. The vascular network component is generated first in the dedicated Vascular Network Channel (VNC). As the vascular network forms, individual lumens form in the area between each posts on the Vascular Channel (VC) for VNC bound media. Neural cells are applied to the Neural Channel side of the nascent vascular network. (“Neural channel”, NC) of the VNC (2, 3). (**b**) The process of platform generation. The perfusable vascular network is formed over a 3-day period via vasculogenesis protocol. The lumen of the vascular network is not yet open to the VC at this time (1). A suspension of freshly isolated neurons and astrocytes is loaded into the NC side of the VNC by applying the cell suspension to the NC media channel and tilting the device in such a way that flows the neurons and astrocytes towards the VNC posts and causes them to settle on the VNC/NC border posts (2). The VC and NC media channels are supplied with their respective optimized media, allowing for the formation of the BBB tissue within 5 to 7 days (3).

The blood-brain barrier (BBB) is a part of the neurovascular unit (NVU) that exist as a complex of blood vessels, astrocytes and neurons^1, 2^. Structurally, the BBB has a direct interface via astrocytic endfeet anchored in the basal lamina of the adjacent capillary wall (Fig. 1a)^3, 4^. At the interface, many transport channel proteins, such as aquaporin 4, facilitate exchange between the astrocytic endfeet and the capillary network^5^. With the complex network of transport channels between the astrocytes and the capillary network, the BBB selectively restricts exchange of blood-bound substrates between the brain and the rest of the circulatory system^6, 7^.

As the BBB is a major obstacle to pharmacologically active substances when targeting the brain, it is the subject of much experimentation and study with regards to CNS drug delivery. Conventional experimentation is generally done *in vivo* on lab animals, which are both expensive and difficult in terms of animal upkeep and purchasing costs, equipment, reagents, and observation^8^. Due to the high entry barrier and costs of current conventional *in vivo* based modeling, the development of a suitable *in vitro* model platform is urgently needed. Transwell platforms, capable of co-culturing segregated cells in micropore membrane separated compartments, are one of the representative platforms for disease modeling and drug screening^9–12^. Booth *et al*. proposed a microfluidic device incorporating Transwell like micron pores into an integrated *in vitro* BBB platform^13–16^. Further studies have also established the capability of generating chemical gradients and cell patterning within membrane integrated microfluidic devices^17, 18^. Recently, Adriani *et al*. proposed a platform of seeding three-dimensional tissue cultures in a ECM hydrogel to produce a structural *in vitro* BBB model^19, 20^. All of the aforementioned BBB platforms are limited by the inability to generate *in vivo* like direct interfaces between astrocytes and a vascular network, do not exhibit the characteristic low permeability of the vascular networks, and are unable to independently treat the inner and outer lumen of the vascular network independently.

The proposed platform employs a system of two separate media microchannels to independently emulate highly localized internal and external vascular microenvironments. The two media microchannels, designated as the Vascular Channel (VC) and the Neural Channel (NC), supply their respective co-culture tissues independently of one another, and can serve as the microenvironment of the outside and the inside of the BBB respectively. The VC connects to the inner lumen of the vascular network, while the NC supplies the neural cells directly attached to the vascular network. The Vascular Network Channel (VNC) generated by this platform exhibits direct contact between neural and vascular tissues and a corresponding low permeability characteristic of *in vivo* BBB (Fig. 1a), suggesting that the dual-channel co-culture method has broad applications in vascular and neural tissue network construction.

## Results

The proposed platform was designed to generate *in vitro* three dimensional co-culture tissues that emulate BBB form and function. In order to generate analogous *in vitro* microenvironmental conditions in both the vascular and neural locales, it was necessary to determine suitable media compositions with optimized quantities for each cell type utilized. Vascular-astrocytic contact areas and vascular network permeability were quantified as metrics to determine media suitability for *in vivo* like co-culture conditions.

### The effect of medium in the neural cell culture

**Figure 2:**
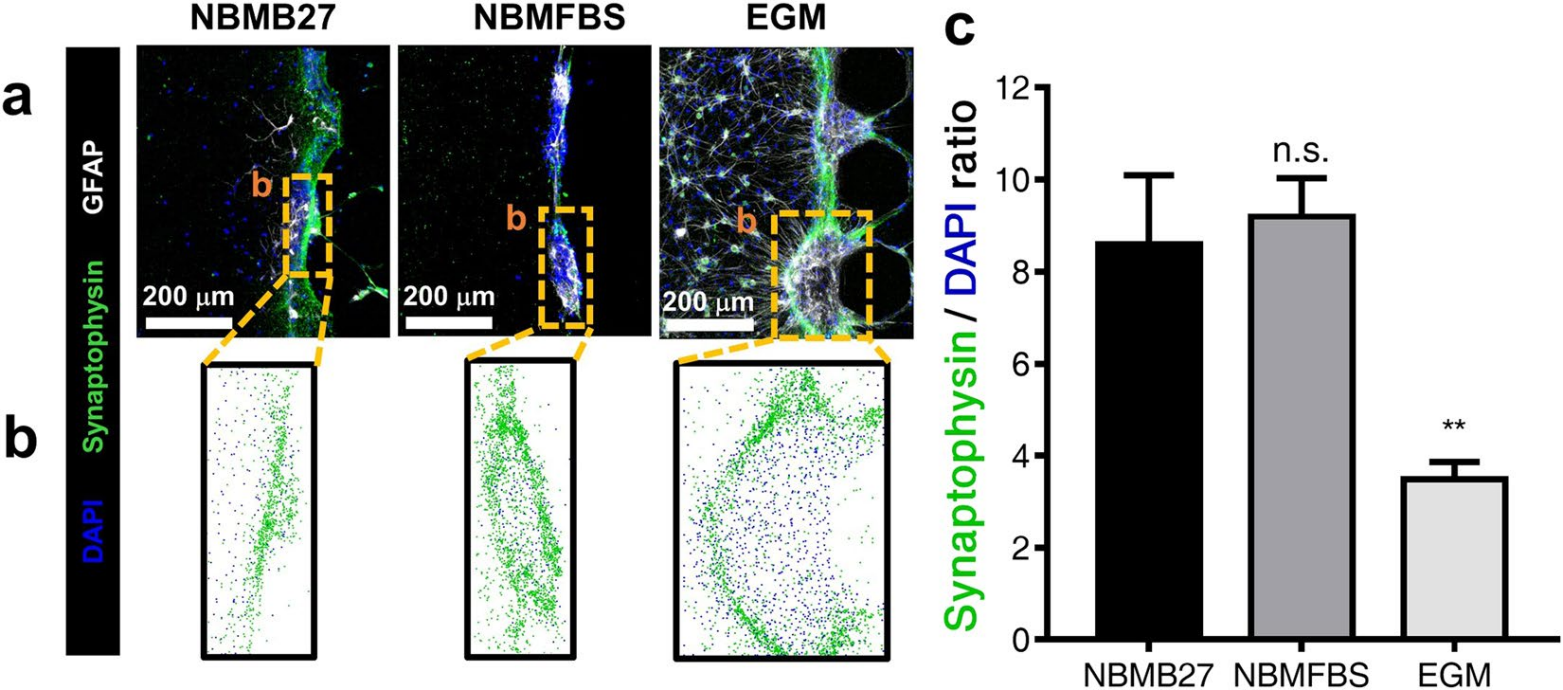
Comparison of three neuron cultures in NBMB27, NBMFBS, and EGM for assessing the effect of FBS in neural cultures. (**a**) Immunostained images of each experimental medium culture. (**b**) The total number of nuclei and synapses identified in each culture. As the neurons were grown in a mass against the fibrin hydrogel wall and do not migrate, synaptic connections were measured at the cell mass. (**c**) The per neuron average of synaptic connections value for NBMFBS was found to be similar to that of cultures grown in NBMB27 control. EGM supplied neuronal cultures show a drastic decrease in synaptic connectivity. All data show mean ± SEM. Statistical analysis student’s t-test. **p < 0.01. 12 ROI for each condition were used for analysis.

While typical neuron cultures are supplied with serum-free B27 (NBMB27**)** neurobasal media, the necessity to introduce serum into the system for the sake of the co-cultured endothelial cells warranted further investigation^21, 22^. Furthermore, as the inclusion of serum in neurobasal media is atypical, it was paramount to determine the potential effects of serum in neural tissue, prior to use. In order to quantitatively assess the effects of serum in neuron culture medium, several characteristics of neuron morphology were selected and observed in an experimental scheme. The ability to form synaptic connections between neurons, as both an objectively quantifiable variable and as a vital characteristic of neurons, served as a suitable candidate for observation. Relative differences in synaptic connections between neurons in serum-free control and various serum containing intervention group media in neuron cultures were quantified and assessed^23, 24^. Neural cells of cortex origin were attached to fibrin hydrogels within test platform microfluidic chips and mono-cultured in different growth media, supplied through both VC and NC channels (Fig. 2a). The supplied media were formulated in the following categories: Neurobasal media (NBM) with FBS; Neurobasal media without FBS; and endothelial growth medium (EGM). For each experimental group, the ratio of synaptic connections to the number of neurons in the cell mass area between microposts (Fig. 2b) were taken as a metric. The experimental media formulations are as follows: NBM with B27 supplement (NBMB27); NBM with 2% FBS (NBMFBS); EGM with 2% FBS. B27 was determined to serve an analogous function in NBM as FBS, and therefore no medium was tested with both B27 and FBS. The resulting data was as follows: NBMB27 8.60 ± 1.50; NBMFBS, 9.20 ± 0.84; EGM, 3.49 ± 0.37. Both NBMB27 and NBMFBS treatment groups exhibited near equivalent synaptic connection per neuron values, while the EGM treatment group was observed to have much lower values (Fig. 2c). As demonstrated by the equivalent per-cell synaptic generation ratio values between NBMB27 and NBMFBS, it was determined that FBS was suitable for use in the proposed co-culture platform.

In addition to diminished synaptic generation, EGM cultured astrocytes were observed to migrate into the adjacent blank ECM (fibrin hydrogel). Neither NBMB27 nor NBMFBS cultured neurons exhibited migration of this nature. As the inclusion of VEGF in EGM is one major difference between EGM and NBM, and it has been reported that VEGF-A has been associated with the increased proliferation and migration of astrocytes, it was postulated that the VEGF in EGM was the cause of the observed EGM cultured astrocyte migration^25, 26^.

**Figure 3:**
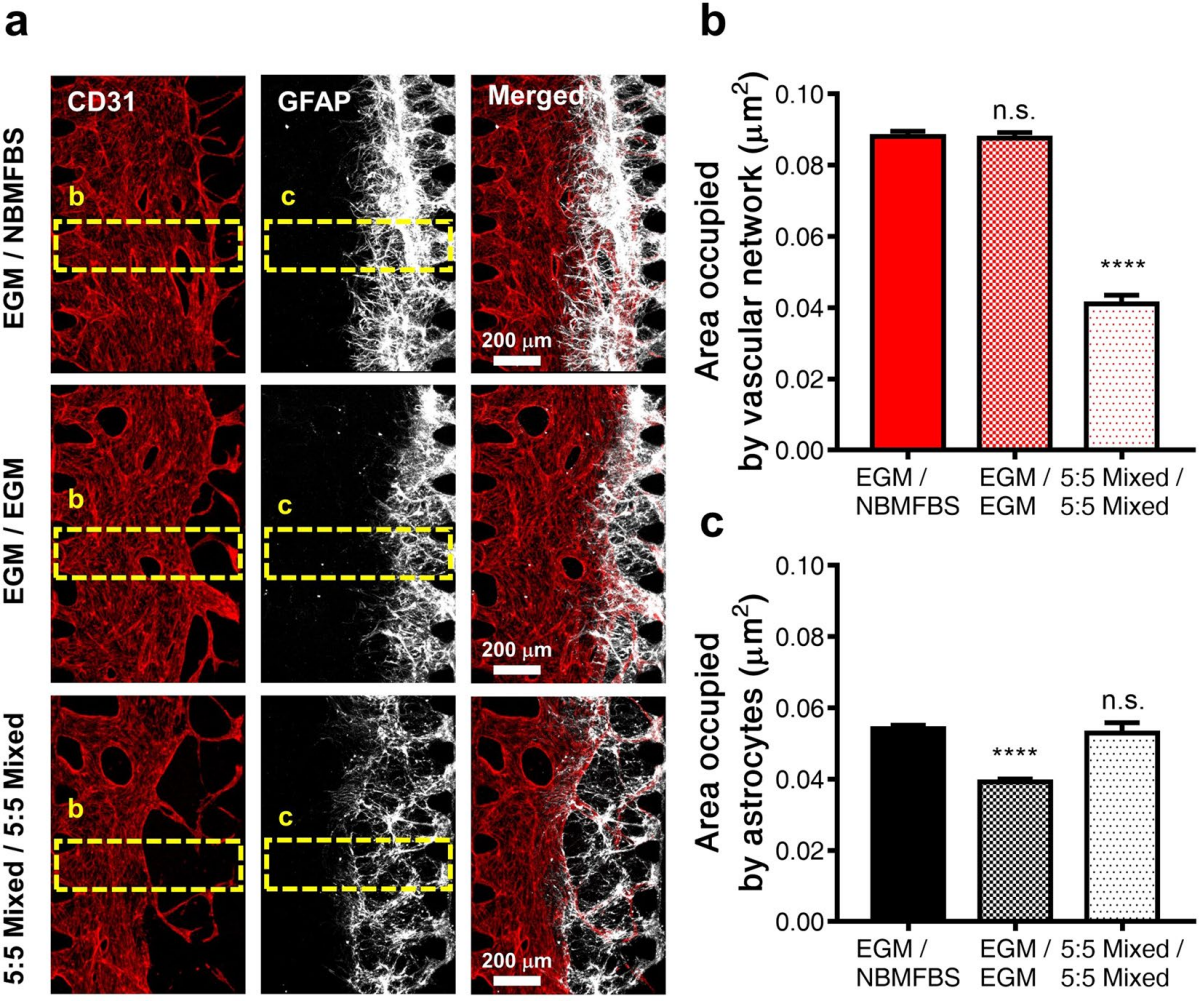
Comparison of vascular network and astrocyte areas in experimental medium co-culture conditions. (**a**) CD31 stained vascular networks shown in red, GFAP stained astrocytes shown in white for all three experimental medium compositions (annotated in VC/NC supplied channels): EGM/NBMFBS; EGM/EGM; 5:5 mix of NBMFBS:EGM for both channels. Vascular network bound media was supplied through the Vascular Channel (VC), and neuron bound media was supplied through the Neural Channel (NC). In the presence of EGM, astrocytes exhibited increased proliferation and migratory behavior. Astrocytes were observed to migrate from the initial neural cell suspension injection zone to the border of the vascular network. (**b**) Average area occupied by the vascular network between a pair of parallel microposts. In cultures where VC was supplied with ECM, (EGM/NBMFBS and EGM/EGM), vascular network areas were roughly equivalent. (**c**) Average area occupied by astrocytes between a pair of parallel microposts. Cultures supplied with NBMFBS via the NC, exhibited the highest astrocytic areas. All data show mean ± SEM. Statistical analysis student’s t-test ****p < 0.0001. 51 ROI for each condition were used for analysis.

To confirm the pro-proliferative and pro-migratory effect of VEGF-A on neurons, separate experimental schemes of neurons in NBMB27 with VEGF-A, and NBMFBS with VEGF-A were cultured. In both cases, neurons cultured entirely in media supplemented with VEGF-A exhibited both increased proliferation and a very brief period of migratory behavior compared to the baseline of VEGF-A absent cultures. Increased proliferation was monitored by means of GFAP stain imaging (Supplementary Fig. 1). When a gradient of VEGF was established in further co-culture experimentation which will be addressed in the next section, astrocytic migration was observed in drastically higher degrees than in co-cultures with a uniform VEGF concentration (Fig. 3c and Supplementary Fig. 3).

### The effect of medium in the co-culture with vascular network and neural cells

**Figure 4:**
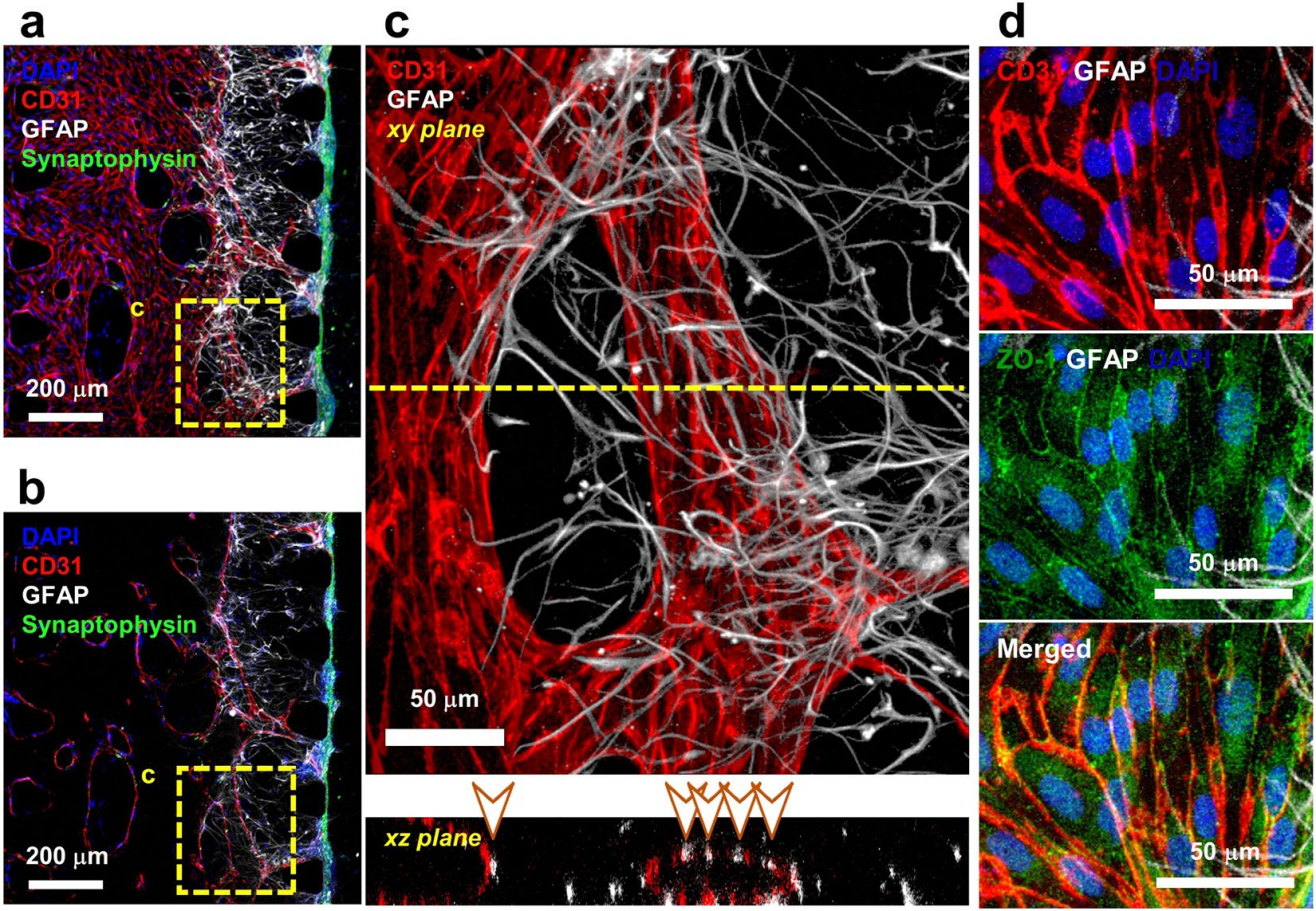
Immunostained imaging of the vascular network - astrocyte interface. (**a**) Astrocytic migration from the NC side neuron-astrocyte loading suspension follows the VEGF gradient towards the VC, and stops after directly interfacing with the vascular network. The neuronal synaptic network is clustered to the right of the fibrin gel, adjacent to the astrocyte-vascular interface. (**b**) An intermediate section layer of the NVU platform. Vascular network lumens form between each VC facing micropost, with each opening occupying the space of the entire post gap. The vascular network is perfusable from the VC, but is not connected to the NC. (**c**) Imaging of the direct vascular network-astrocyte interface. Confocal microscopy confirmed direct contact on two axes, with contact points indicated in the xz plane render via arrow points. (**d**) Vascular network - astrocyte contact confirmed through immunostaining for ZO-1 tight junction protein expression by CD31 positive endothelial cells.

As it was with the investigating the impact of new supplementary substrates in neural cultures, the same consideration was warranted for endothelial cells. To test the effect of NBMFBS on the vascular network, a co-suspension of HUVEC and fibroblasts in fibrin were loaded first on the VC side of the device. Vascular networks formed from the seeded HUVEC and LF after three days of incubation, although the lumen of the network did not open towards the media channel at this time. Neural cells are then seeded on the surface of the fibrin hydrogel surface into the neuron growth chamber (Fig. 1b). At this time, the vascular network is displaced away from the NC, and the vascular lumen is connected to the VC. Astrocytes have also been observed migrating towards the vascular network, stopping only when contact with the EC is made. In all cases, astrocytic migration was observed to stop at the vascular network (Fig. 3a and Supplementary Fig. 2). ZO-1 immunostaining was used to confirm the presence of BBB tight junctions along the interface regions between astrocytes and the vascular network (Fig. 4d)^27^.

At all tested concentrations, in the presence of NBMFBS, vascular networks were observed to contract and narrow. Conversely, the presence of EGM was observed to preserve typical vascular network morphology. The resulting measurements per media composition are as follows (medium in the VC/medium in the NC): EGM/NBMFBS, 0.0881 ± 0.0015 μm^2^; EGM/EGM, 0.0876 ± 0.0016 μm^2^; 5:5 mixed/5:5 mixed, 0.0411 ± 0.0025 μm^2^ (Fig. 3b). Results confirm that EGM is indeed the superior growth medium for optimal vascular network growth and maintenance. Different media conditions were also observed to influence the degree of astrocytic migration. To quantify astrocytic migration under the tested media composition profiles, the area of astrocyte migration was observed in each experimental group. The observed astrocytic migratory areas by media composition are as follows (medium in the VC/medium in the NC) EGM/NBMFBS, 0.0542 ± 0.0010 μm^2^; EGM/EGM, 0.0393 ± 0.0008 μm^2^; 5:5 mixed/5:5 mixed, 0.0529 ± 0.0029 μm^2^ (Fig. 3c). The data suggests that NBMFBS supplied to the NC results in a larger area of astrocytic migration than when EGM is supplied to NC. To reiterate, the supply of EGM to the VC appears to aid in maintaining good vascular morphology, and the supply of NBMFBS to the NC appears to promote astrocytic migration. In co-cultures supplied with NBMFBS to the NC and EGM to the VC, astrocytic and vascular network overlap was the highest amongst all other tested co-cultures. Given the VEGF-A mediated promotion of neuron proliferation and migration as observed previously, it was hypothesized that the generation of a VEGF gradient within the fibrin hydrogel may have played a part in the increased vascular network and astrocytic overlap. To confirm the hypothesis, co-cultures supplied with media containing VEGF-A on one side through the VC, and VEGF-A absent media through the NC were compared with co-cultures supplied with exclusively VEGF-A supplemented and VEGF-A absent media. Co-cultures with a VEGF-A gradient exhibited a much higher degree of vascular/astrocytic overlap than in non-gradient co-cultures. In order to visualize the gradient of VEGF-A side media with a VEGF-A/nonVEGF-A medium supplied co-culture, FITC-dextran was added to the VC prior to the opening of the vascular lumen to the channel. A linear gradient of FITC-dextran was observed in the fibrin hydrogel originating from the VEGF supplied media channel (Supplementary Fig. 3).

Due to the importance of confirming the direct contact between astrocytes and the vascular network, overlap areas were imaged via immunofluorescence staining high-resolution confocal microscopy. Because of the three dimensional nature of the platform, it was difficult to confirm direct contact between astrocytes and the vascular network from top-down imaging alone, as tissue formation may have occupied the same area from a top down view, but may have grown at different elevations of the channel and thus did not physically come into contact. Therefore, neurovascular interfaces were visually confirmed through imaging on a two-axis cross-sectional basis to ensure both top-down and lateral contact. (Fig. 4 and Supplementary Fig. 4). This phenomenon was observed in every medium condition (Supplementary Fig. 5). In addition, aquaporin 4 channels were observed only where direct neurovascular contacts formed. (Supplementary Fig. 6).

### The effect of medium in permeability

**Figure 5:**
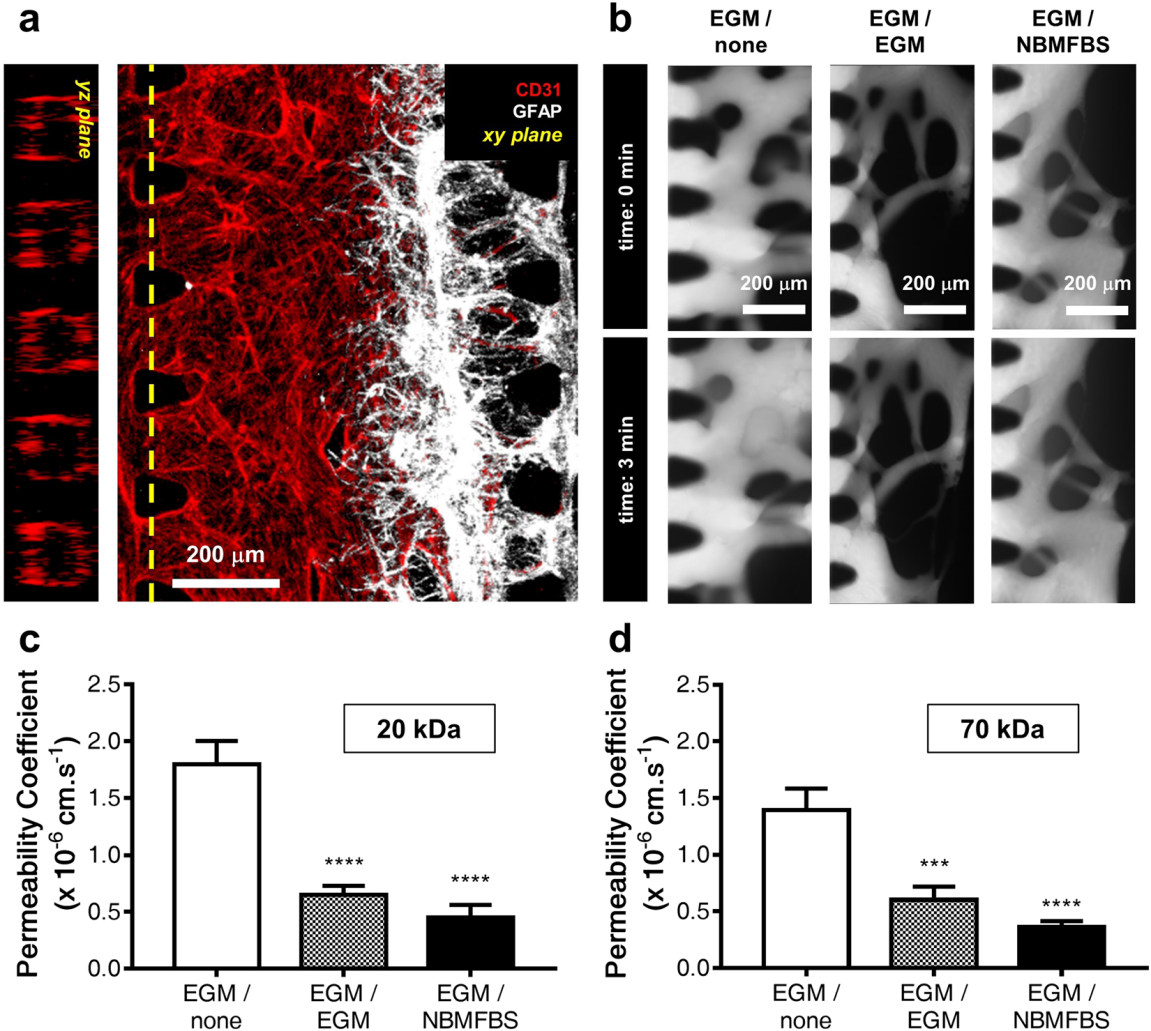
Vascular network - astrocyte complex permeability compared across the three experimental co-culture medium compositions. (**a**) Immunostained imaging of the vascular network and the astrocytic interface confirms that vascular lumens cover all of the areas between VC side microposts. This means that any substances introduced through the VC into the vascular network can be assumed to be flowing inside the vascular network and not in the fibrin hydrogel. (**b**) Time-lapse microscopic photographs at various medium conditions with 70 kDa FITC-dextran. (**c**) Results of permeability experiments with 20 kDa FITC-dextran. (**d**) Results of permeability experiments with 70 kDa FITC-dextran. All data show mean ± SEM. Statistical analysis student’s t-test. ***p < 0.001, ****p < 0.0001.

Permeability was quantified for flow exclusively through the lumen of the vascular network to the astrocytes. The vascular network was generated by means of seeding endothelial cells along the VNC. After the formation of the network, the space in between each micropost became the entry port of a single vascular lumen which stretched from post to post. (Fig. 5a and Supplementary Fig. 7). Any substances introduced into the entrance of the vascular lumen would move into the vascular network through the lumen exclusively, and not through the fibrin hydrogel. As the formation of fully perfusable and closed lumen only occurred in co-cultures with EGM supplied VC, permeability tests could only be done on EGM supplied VC platforms. After introducing 20 kDa FITC-dextran into the vascular network via the VC, the amount of leakage outside of vascular network was measured in three conditions (medium in the VC/medium in the NC): only cultured vascular network (EGM/none) and co-cultured vascular network with different medium conditions (EGM/EGM and EGM/NBMFBS). Fluorescence in the vascular network was expressed immediately after introduction of FITC-dextran through the VC. The intensities of fluorescence inside and outside of vascular network were observed in real time (Fig. 5b). The permeability value of the EGM/none condition was higher than that of EGM/EGM condition. In the case of the co-cultured platform, the permeability value of the EGM/NBMFBS condition was lower than that of EGM/EGM condition. (Permeability coefficients with 20 kDa FITC-dextran, EGM/none (n = 9), 1.85 ± 0.20 × 10^−6^ cm/s; EGM/EGM (n = 14), 0.65 ± 0.08 × 10^−6^ *cm/s*; EGM/NBMFBS (n = 13), 0.45 ± 0.11 × 10^−6^ cm/s; Permeability coefficients with 70 kDa FITC-dextran, EGM/none (n = 12), 1.39 ± 0.19 × 10^−6^ cm/s; EGM/EGM (n = 12), 0.60 ± 0.12 × 10^−6^ cm/s; EGM/NBMFBS (n = 20), 0.36 ± 0.05 × 10^−6^ cm/s (Fig. 5c and d). Comparison of permeability coefficients with other *in vitro* BBB models is summarized in Table 1.

**Table 1:** Permeability coefficients in various *in vitro* BBB models measured by 20 kDa and 70 kDa FITC-dextran.

Model	20 kDa (10^−6^ cm/s)	70 kDa (10^−6^ cm/s)	References
*In vivo*	0.24	0.15	Yuan, W. *et al*. (2009)^32^
Transwell	—	0.16	Li, G. *et al*. (2010)^29^
Microfluidic, membrane	~2	~0.7	Booth, R. *et al*. (2012)^14^
Microfluidic, hydrogel	—	~10	Adriani, G. *et al*. (2017)^19^
This work	0.45	0.36	

## Discussion

The blood-brain barrier, a critical part of the NVU, is formed from a three dimensional monolayer of endothelial cells directly interfaced with astrocytes via a system of capillaries and characterized by low permeability^3^. This study proposes a platform which is capable of generating a three dimensional BBB microfluidic platform which presents both structural and functional properties of the BBB *in vivo*. The vascular network generated in the proposed BBB platform is comprised of intact and perfusable monolayer capillaries and directly contacts astrocytes in a manner resembling the NVU^28^.

Astrocytes, which are an important component of the BBB, plays a role in controlling capillary features and BBB permeability^29^. However, it is known that angiogenesis is inhibited if astrocytes and endothelial cells are plated at the same time^30^. Therefore, the cell plating procedure in this study emulated *in vivo* brain development in that blood vessels are generated before neuronal seeding^31^.

In the neural cell culture of this study, the robust proliferation and the migration of astrocytes were rarely observed with exclusively NBMFBS supplied media, but observed in EGM supplied cultures (Fig. 2a). VEGF-A was also observed to promote the proliferation and the migration of astrocytes (Supplement Fig. 1). In contrast, EGM/NBMFBS supplied co-cultures exhibited larger astrocytic areas than in EGM/EGM supplied co-cultures (Fig. 3c). The resulting data suggests that the gradient of VEGF-A in the fibrin hydrogel promotes astrocytes to proliferate and migrate toward the VC in EGM/NBMFBS supplied co-cultures. In EGM/EGM supplied co-cultures, the proliferation and the migration of astrocytes toward the VC are less pronounced due to the lack of VEGF-A gradient. Therefore, it was presumed that overlapped large area between vascular networks and astrocytes was made due to VEGF-A gradient within fibrin hydrogel at EGM/NBMFBS condition. The increase of the overlapped area between vascular network and astrocytes indicates that the total area of direct contact between vascular network and astrocytes is increased. It is known that vascular network expresses better BBB features when direct contact area between vascular network and astrocytes increases^30^. As a result, it could be assumed that the permeability at EGM/NBMFBS condition, which is similar to *in vivo* BBB^32^, is lower than at EGM/EGM condition due to the increased direct contact area. Consequently, it is reasonable that the permeability decreases as overlapped area between vascular network and astrocytes increases.

One limitation of this study is the use of umbilical cord endothelial cells rather than brain derived endothelial cells. However, the study indicates that the platform can present structural and functional equivalency with *in vivo* BBB while still using HUVEC. Akiyama *et al*. also showed that HUVEC can be formed into the BBB when grafted into an *in vivo* mouse brain^33, 34^. Likewise, Hayashi *et al*. published that *in vitro* co-culture with astrocytes and HUVEC presents brain-type glucose transporter (GLUT-1) expression which is one of the characteristics of the BBB^30^.

Neuropharmacological research is severely hampered by BBB impermeability, as drugs must first pass the BBB before gaining access to the brain^35–37^. *In vivo*-like *in vitro* NVU platforms may promote research on brain disease models, and allow for an easier means of pharmacologically targeting the brain. Although the proposed co-culture method could be applied to other tissues co-cultured with naturally lumenized 3D vascular networks, the ultimate goal of this publication is to provide a complete *in vitro* NVU platform consisting of naturally lumenized 3D vascular networks, astrocytes with the BBB structure, and synapses on neurons. The NVU has many types of intercellular interactions, such as the interactions between endothelial cells and astrocytes, endothelial cells and microglia, and astrocytes and neurons^3, 38, 39^. The proposed platform cannot be called a complete NVU model as of yet, as it can only model the interactions between endothelial cells and astrocytes. Further studies are needed in order to expand the platform to an all-inclusive NVU on a chip, which would greatly accelerate the fields of neuroscience and neuropharmacology.

## Methods

### Microfluidic device fabrication

Microfluidic devices are fabricated by soft lithography, which utilizes a master device to create multiple soft molds. The master with embossed structure of negative photoresist SU-8 (MicroChem) was developed by photolithography on a 4-inch silicon wafer. A 10:1 (w/w) Polydimethylsiloxane (PDMS, Sylgard 184, Dow Corning) and curing agent was poured on the master and degassed in a vacuum chamber for removing bubbles and thermally cured to obtain a negative replica molds. Using biopsy punch (6 mm) and sharpened blunt syringe needle (0.5 mm), medium reservoirs and hydrogel injection parts were punched out. The PDMS molds and glass coverslip, which are treated with oxygen plasma to form covalent bonding between them, were cleaned with residue-free tape and nitrogen gas air gun. After bonding, the device was followed by the incubation in an 80 °C dry oven at least 48 hours to maintain hydrophobic condition. The devices were sterilized by UV irradiation before experiment.

### Cell culture

Human umbilical vein endothelial cells (HUVECs, Lonza) were cultured in endothelial basal medium-2 supplemented with EGM-2 SingleQuots Kit (EGM-2, Lonza) and passages 3 to 5 were used for the experiments. Normal human lung fibroblasts (LFs, Lonza) were cultured in fibroblast basal medium supplemented with FGM-2 BulletKit (FGM-2, Lonza) and passages 5 to 7 were used for the experiments. In terms of HUVECs, cells were cultured for experiments before reaching 80 to 90% of confluent. All cells were incubated in a humidified 5% CO_2_ atmosphere and at 37 °C.

### Cell plating for vasculogenesis in the microfluidic device

Fibrinogen solution was made by dissolving bovine fibrinogen (10 mg/ml, F 8630, Sigma-Aldrich) in Dulbecco’s phosphate-buffered saline (DPBS, Hyclone) and filter sterilized (0.22 μm pore). Then, Mixing with aprotinin (0.15 U/mL, Sigma-Aldrich). HUVECs and LFs, which are detached from the cell culture dishes by treating 0.25% Trypsin-EDTA (Hyclone), were centrifuged and suspended at concentration of 6.7 million cells per ml in EGM-2 medium. The cell suspensions are mixed with the fibrinogen solution at a ratio of 3:1 to yield a final concentration of HUVECs and LFs as 5 million cells per ml, respectively. The mixtures with thrombin (0.5 U/ml, T4648, Sigma-Aldrich) were injected into the center hydrogel micro-channel and side micro-hydrogel channel. After 5 minutes at room temperature, the gel mixtures formed structures and the upper reservoirs in each device were filled with culture medium (EGM-2) and aspirated gently at the lower reservoirs to make the hydrophobic medium micro-channel. Following filling evenly rest of reservoirs with the medium, the devices were incubated at 37 °C and 5% CO_2_. The culture medium was changed into fresh EGM-2 culture medium every 48 hours.

### Cortex neural cell preparation and plating in the microfluidic device

We used Sprague-Dawley embryonic rat (E17) for preparing rat cortical neurons. The rat cortices in trypsin-EDTA solution (Gibco, USA) were incubated at 37 °C water-bath for 12 min. After incubating, the trypsin-EDTA solution took away, and Dulbecco’s modified Eagle’s medium (DMEM, Gibco, USA) with 10% Fetal Bovine Serum was supplied to stop trypsin reaction. The DMEM solution was removed, and Neurobasal medium (Invitrogen) with 2% B27 supplement (Invitrogen, USA), 0.25% GlutaMax (Invitrogen), and 1% penicillin–streptomycin (Invitrogen, USA) was supplied. The cortices were cut into small pieces with 1 ml and 200 µl pipet tip. After this process, the cell suspension was made and filtered by cell strainer. The cell suspension is diluted with neurobasal medium to required concentration, 8 million cells per ml. The 50 µL cell suspension was put into microfluidic device. To settle the cells down to the surface of fibrin hydrogel, the microfluidic device was tilted at 90° degree in 37 °C incubator for 30 min. For VEGF-A test, VEGF-A was added to Neurobasal medium so that the concentration of VEGF-A was 100 ng/ml. All acquisition procedures of biological samples were approved by the Institutional Animal Care and Use Committee of the Seoul National University, and all experiments were conducted in accordance with the relevant guidelines and regulations set by the Committee.

### Immunostaining

Samples were washed at least twice with PBS and fixed in 4% paraformaldehyde (PFA, Thermo) for 15 minutes at room temperature. After fixation, cells were permeabilized with 0.15% triton-X 100 (Sigma-Aldrich) for 20 minutes at room temperature. Samples were then treated with 3% bovine serum albumin (BSA, Sigma-Aldrich) to minimize nonspecific antibody binding and Hoechst 33342 in PBS to stain nuclear for 1 hour at room temperature. After washing with PBS, samples were stained with Alexa Fluor(R) 488 anti-mouse CD31 antibody (monoclonal, Cat. No. 303110, diluted 1:200, Biolegend) as an endothelial cell marker, Alexa Fluor(R) 647 anti-GFAP antibody (monoclonal, Cat. No. 560298, diluted 1: 200, BD phamigen) and anti-synaptophysin antibody (Polyclonal, Cat. No. ab32594, diluted 1: 200, Abcam).

### Synapse number measurement

Synapse images were determined by staining for synaptophysin. Images were taken using a confocal microscope (Olympus FV1000). IMARIS (Bitplane, Switzerland) was used for reconstructing three dimensional images. The cells between micro-posts were analyzed. Using the Spot function of IMARIS, the nucleus of cell above 4.5 µm diameter and the synaptophysin above 1.0 µm diameter were counted.

### Area of vascular network and astrocytes measurement

The 3D reconstructions and cross-sections of the vascular network and astrocytes were determined by using a confocal microscope (Olympus FV1000). Images were analyzed by imageJ. To quantify the area of vascular network, Z-projections of the 3D stacks were obtained and then each image was masked from the backgrounds before measurement.

### Permeability coefficient measurement

A fluorescence image of FITC-dextran diffusing across vascular network was analyzed for calculating the permeability coefficient. After removing all media from medium reservoirs, 50 μl of 20 kDa FITC-dextran solution was flowed into the VC. 70 kDa FITC-dextran solution was treated as the same way. For observation of the permeability of the vascular network, an inverted epifluorescence microscope (Olympus IX81) was used. Images were taken in every 25 second interval. The equation for calculating the permeability coefficient is derived from previous study.

### Statistical analysis

All data were plotted as mean ± SEM. The student’s t-test is used statistically to analyze the different datasets.

## Electronic supplementary material

Supplementary information
