# Supplementary material for: A Low Permeability Microfluidic Blood-Brain Barrier Platform with Direct Contact between Perfusable Vascular Network and Astrocytes
Source: Sci Rep. 2017 Aug 14;7:8083. doi: 10.1038/s41598-017-07416-0 (PMC5556097; doi:10.1038/s41598-017-07416-0)
Supplement: Supplementary file 1 — Supplementary information [file 41598_2017_7416_MOESM1_ESM.pdf]

Supplementary Information

A Low Permeability Microfluidic Blood-Brain Barrier Platform with Direct Contact between Perfusable Vascular Network and Astrocytes

Seokyoung Bang[1], Seung-Ryeol Lee[1], Jihoon Ko[1], Kyungmin Son[1], Dongha Tahk[1], Jungho Ahn[1], Changkyun Im[2] & Noo Li Jeon[1,3*]

[1]Division of WCU (World Class University) Multiscale Mechanical Design, School of Mechanical and Aerospace Engineering, Seoul National University, Seoul, South Korea

[2]BK21 Plus Transformative Training Program for Creative Mechanical Engineers, Seoul National University, Seoul, South Korea

[3]Institute of Advanced Machinery and Design, Seoul National University, Seoul, South Korea

[*]Correspondence and requests for materials should be addressed to N.L.J. (email: njeon@snu.ac.kr)

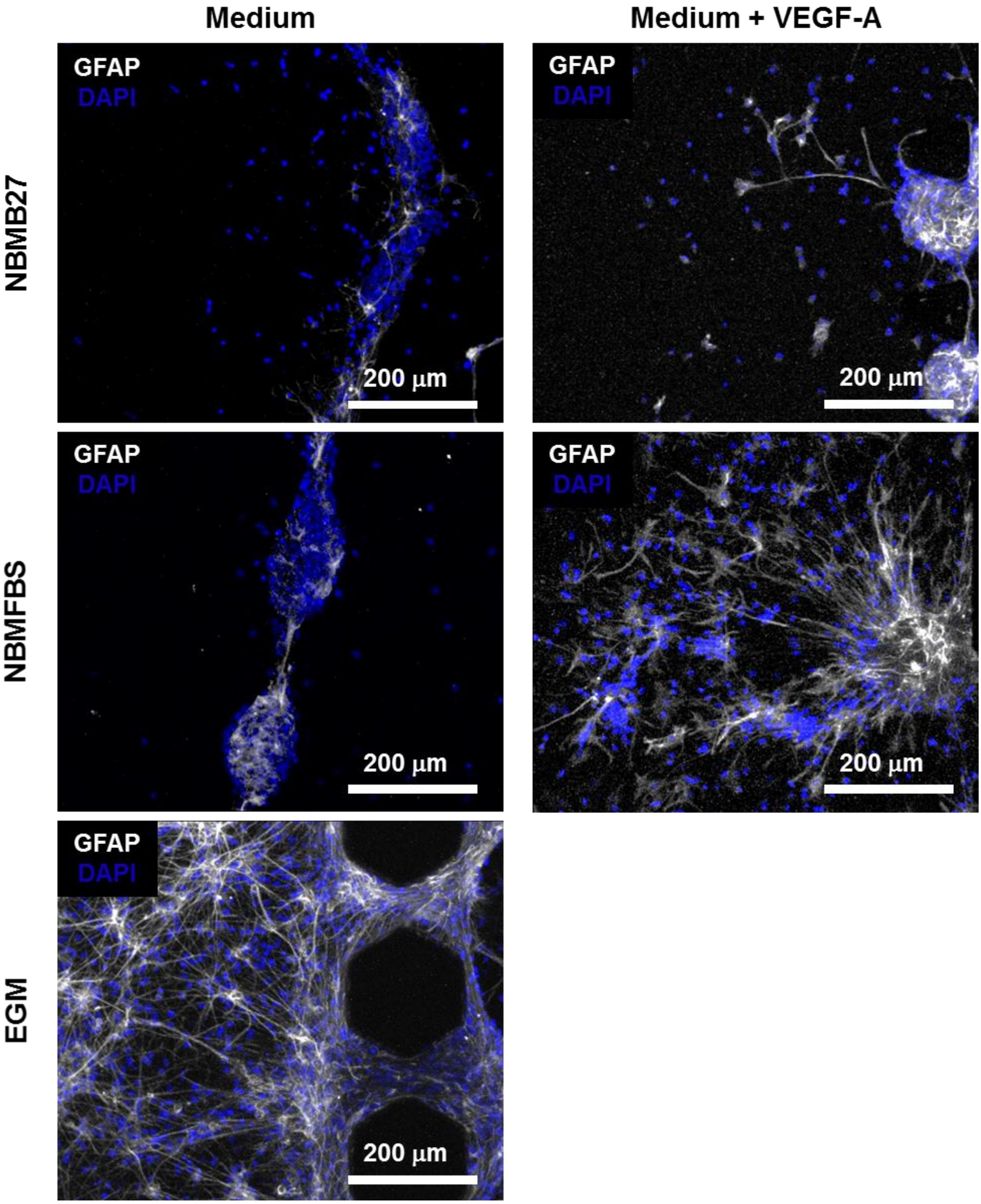

Supplementary Figure 1. The effect of VEGF-A on the proliferation of astrocytes. Unlike EGM, NBMB27 and NBMFBS do not have VEGF. Astrocytes also proliferate prominently in EGM condition but less proliferate in NBMB27 condition and NBMFBS condition. On the other hand, the proliferation of astrocytes is observed when VEGF-A (100 ng/ml) is added to NBMB27 or NBMFBS.

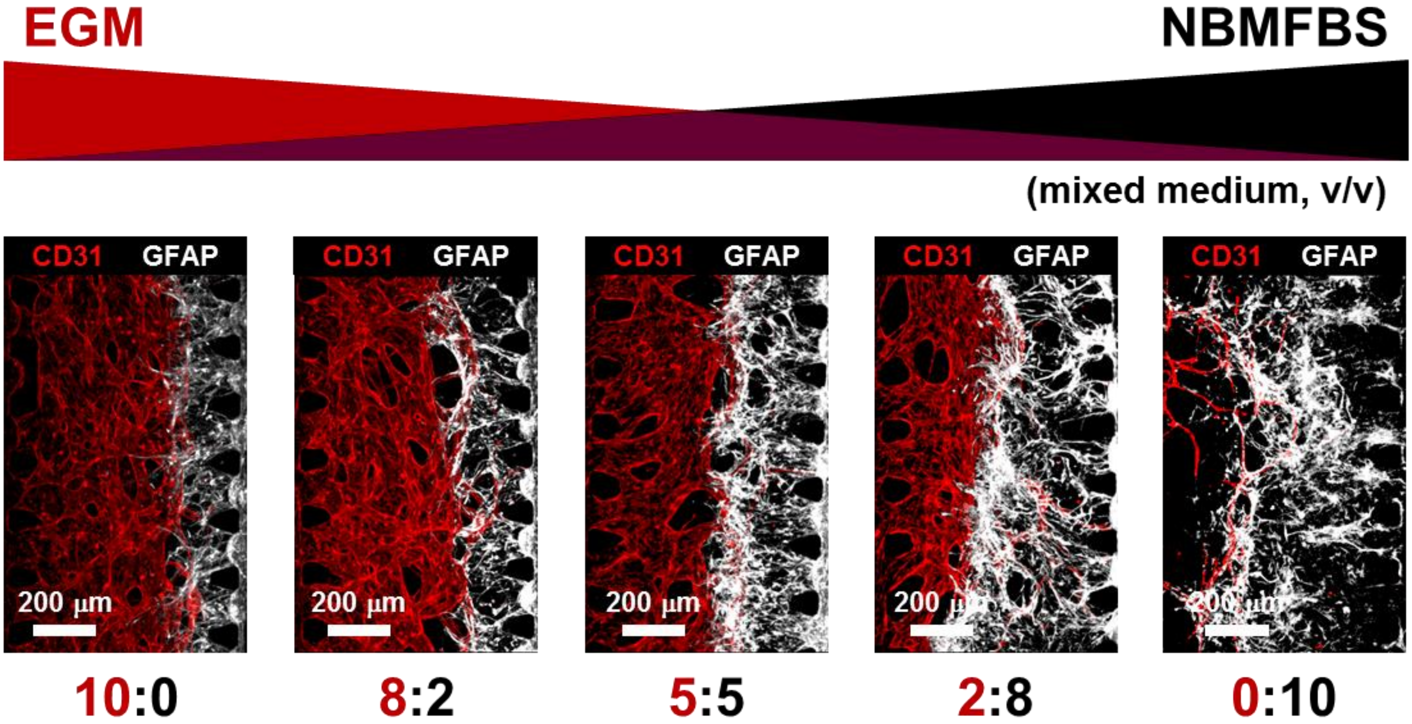

Supplementary Figure 2. Area difference of vascular network and astrocytes under various conditions. In this experiment, the medium in VC and NC is the same. Reduced amount of EGM in the medium micro-channels makes smaller area of the vascular network. However, reduced amount of EGM in the medium micro-channels makes the larger area of the astrocytes.

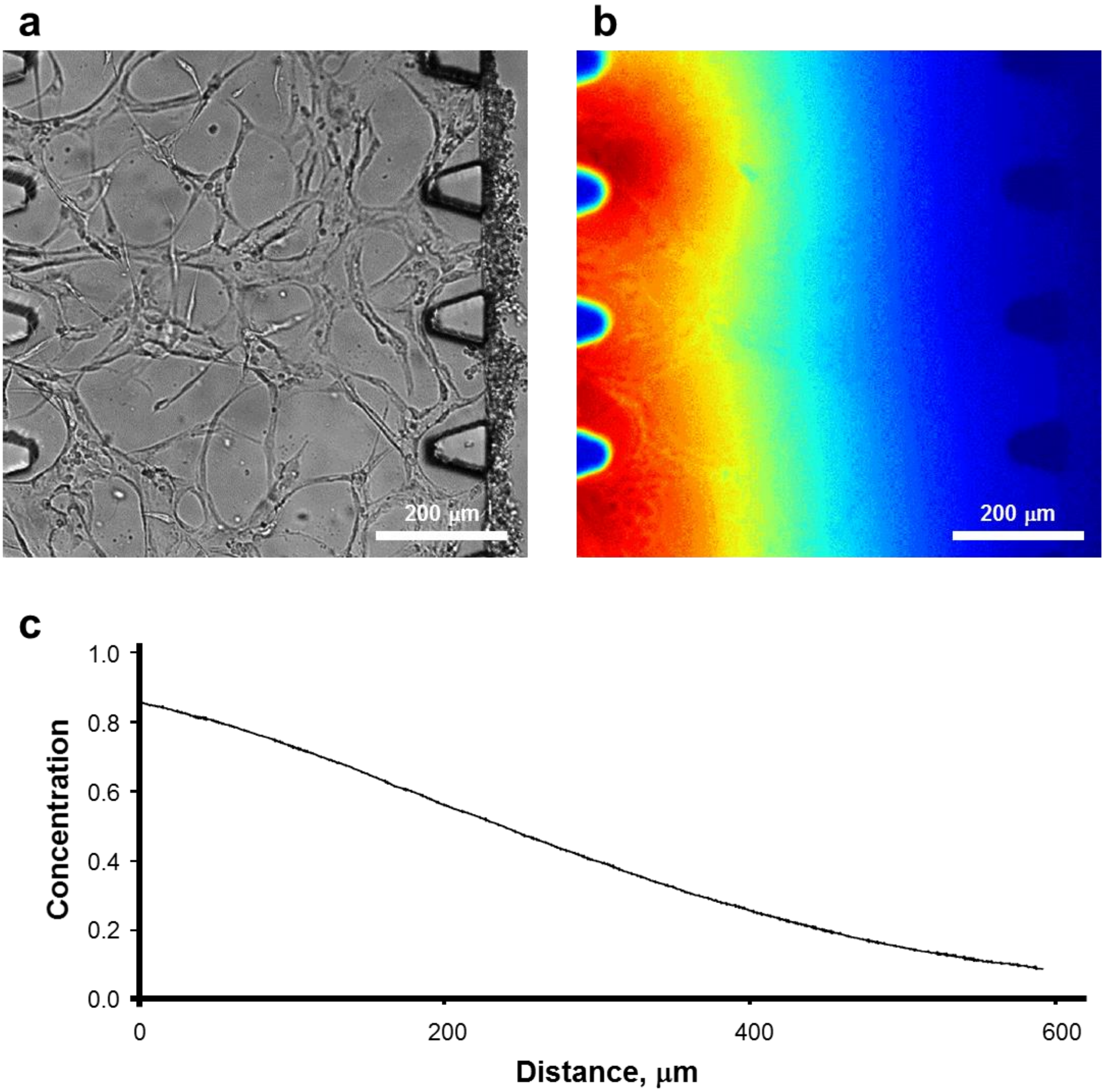

Supplementary Figure 3. Linear gradient inside fibrin hydrogel before lumen of vascular network opens to medium channel. (a) The vascular network was cultured in the fibrin hydrogel for 3 days. The lumen of the vascular network is still not connected to the medium channel. Therefore, the wall surface of the fibrin hydrogel is still maintained. At this point, neural cells are attached to one side of the fibrin hydrogel. FITC-dextran was injected into the medium channel opposite to the neural cell. (b) At this time, FITC-dextran (20 kDa) gradient across the fibrin hydrogel is not affected by the vascular network. FITC-dextran shows a linear gradient in the fibrin hydrogel. (c) Diffusion profile inside of fibrin hydrogel. This diffusion profile was measured 10 minutes after FITC-dextran injection.

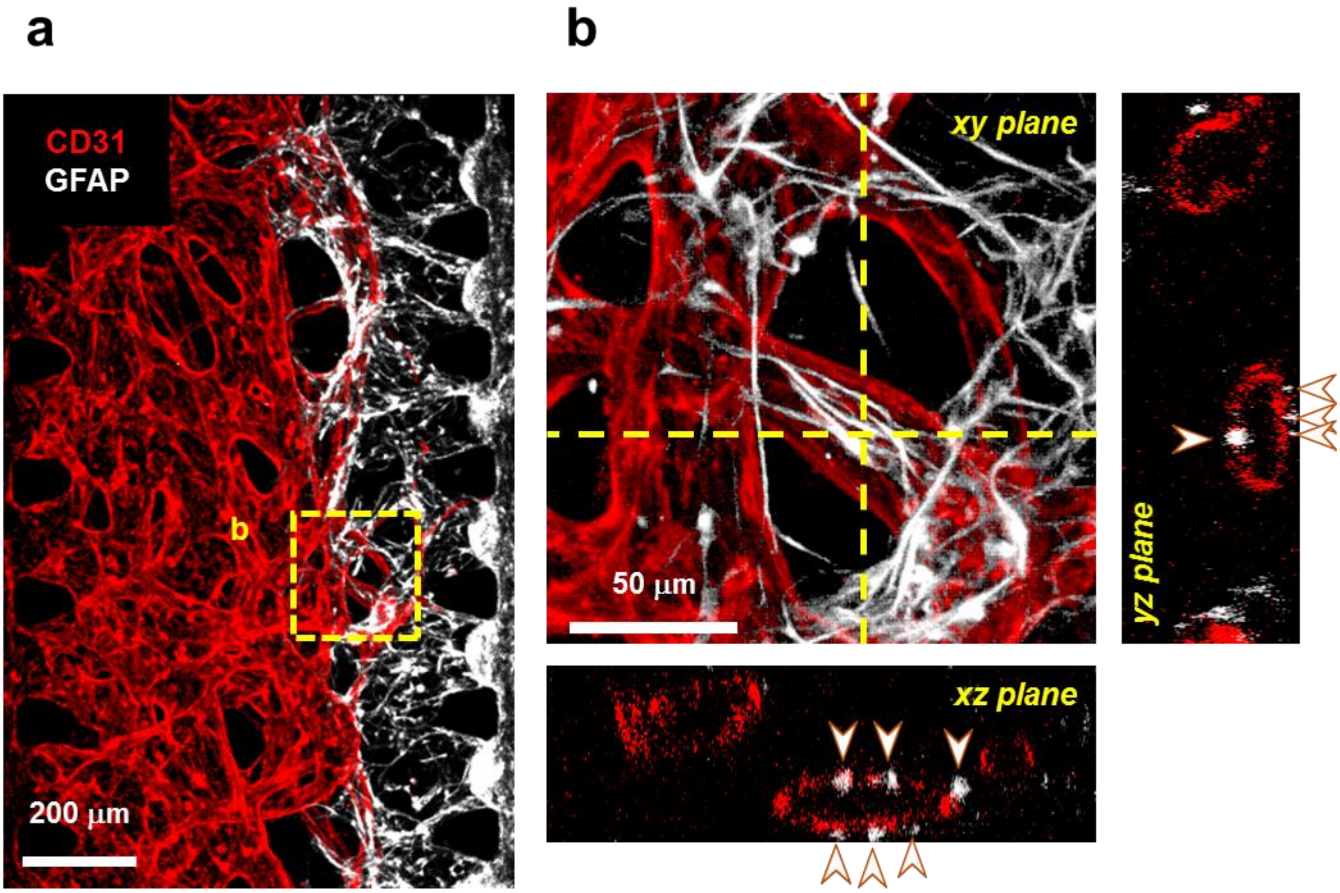

Supplementary Figure 4. Direct contact of the vascular network and astrocytes. A large number of direct contacts between vascular network and astrocytes is observed not only xz plane but also yz plane (arrowhead).

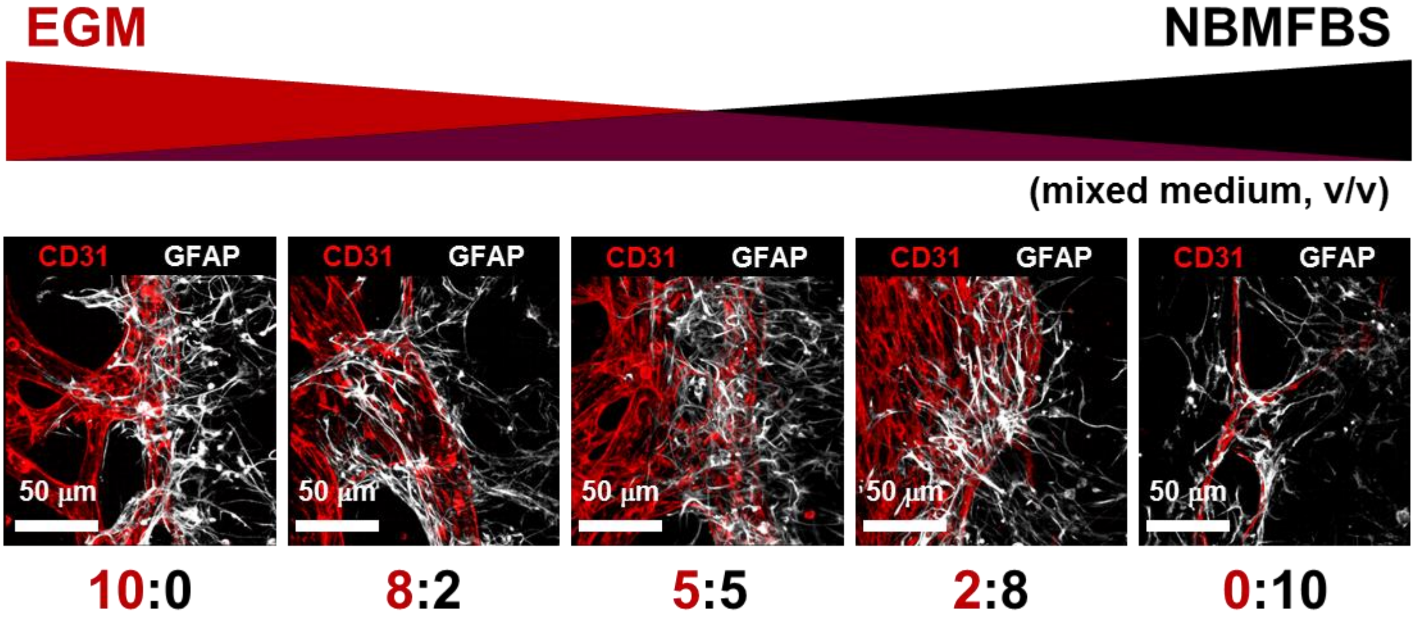

Supplementary Figure 5. Direct contact between vascular network and astrocytes under various conditions. In this experiment, the medium in VC and NC is the same. Although medium conditions are different, a number of direct contacts occurs.

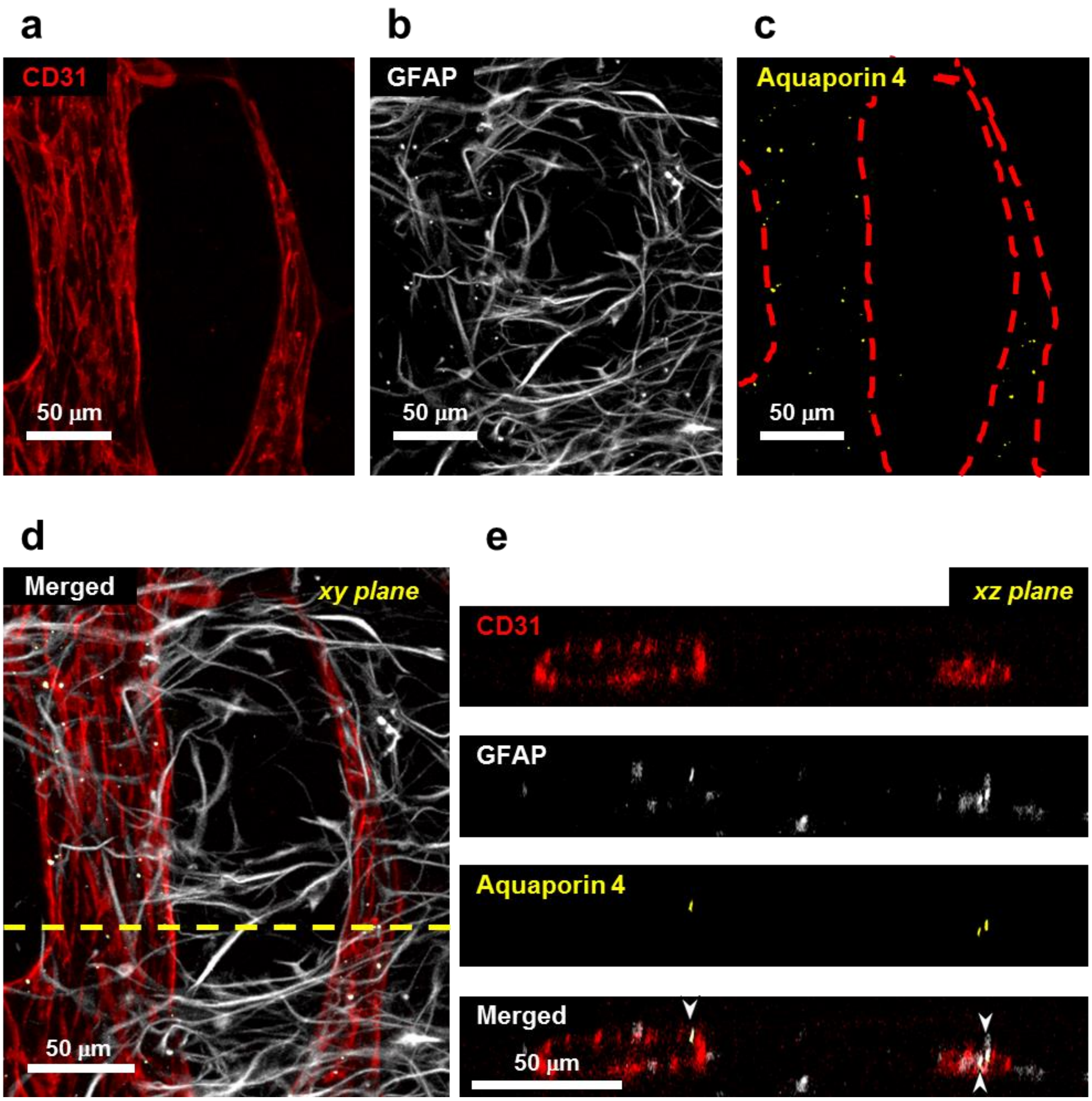

Supplementary Figure 6. Expression of aquaporin 4 at the direct contact between vascular network and astrocytes. (a) Immunostained image of vascular network (b) Immunostained image of astrocytes (c) Immunostained image of aquaporin 4. The red dotted line represents the outline of the vascular network. The aquaporin 4 is mostly expressed on the vascular network. (d) Merged image of vascular network, astrocytes, and aquaporin 4. (e) The aquaporin 4 is expressed at the direct contact between vascular network and astrocytes.

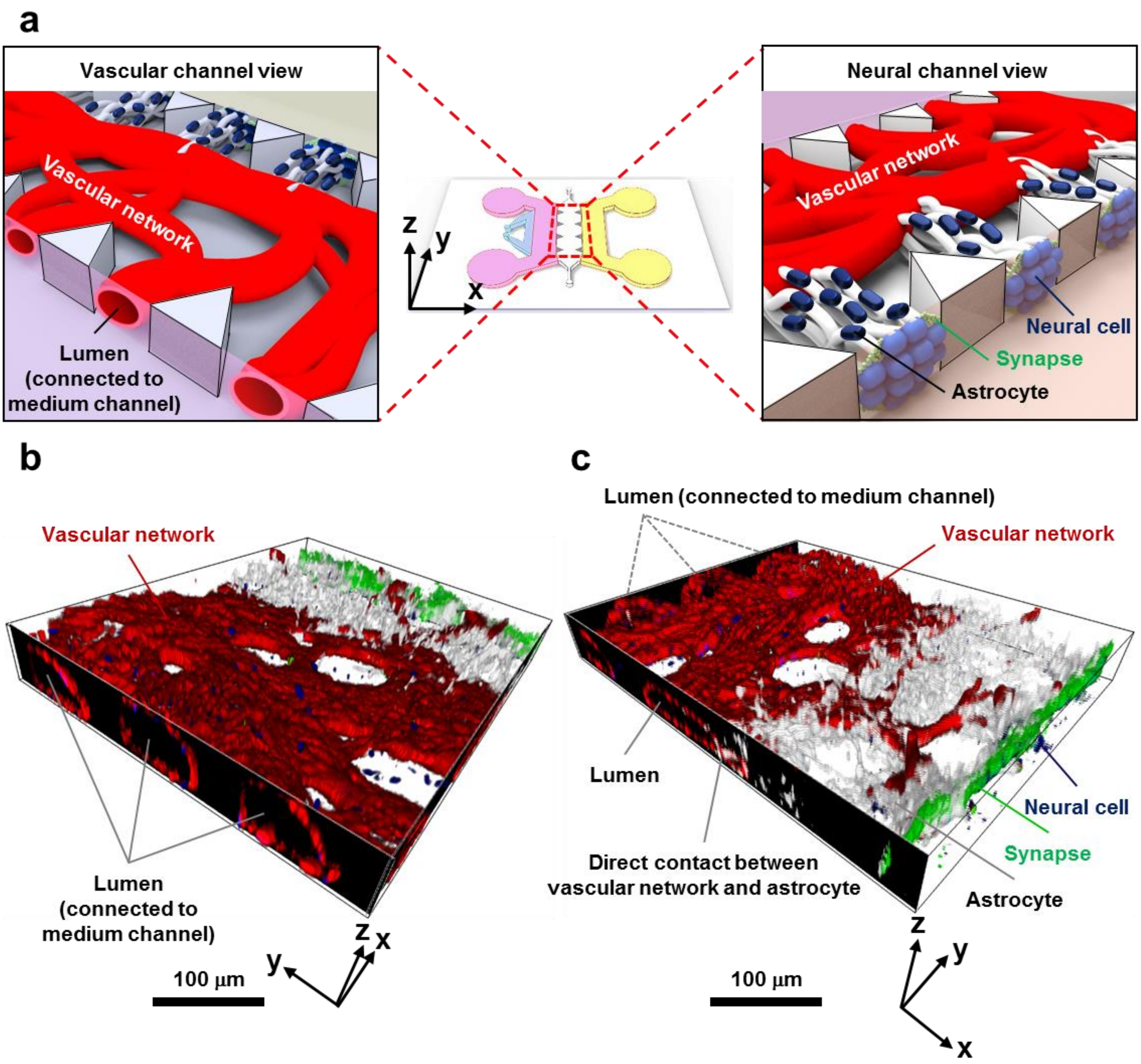

Supplementary Figure 7. The 3D images of immunostained NVU platform. (a) Scheme of the microfluidic in vitro 3D NVU platform including BBB. (b) 3D NVU platform viewed from the direction of vascular channel. There is lumen between micro-posts that connects to the inside of the vascular network. (c) 3D NVU platform viewed from the direction of neural channel. Synapse is expressed at the area in contact with the neural channel. Also, as can be seen from the xz sectioned plane, the lumen of the vascular network is well maintained. These 3D images are reconstruction of figure 4.
